# A dual death/survival role of autophagy in the adult ovary of *Lagostomus maximus* (Mammalia- Rodentia)

**DOI:** 10.1371/journal.pone.0232819

**Published:** 2020-05-29

**Authors:** Noelia P. Leopardo, Mariela E. Velazquez, Santiago Cortasa, Candela R. González, Alfredo D. Vitullo

**Affiliations:** 1 Centro de Estudios Biomédicos Básicos, Aplicados y Desarrollo CEBBAD, Universidad Maimónides, Buenos Aires, Argentina; 2 Consejo Nacional de Investigaciones Científicas y Técnicas, CONICET, Buenos Aires, Argentina; Faculty of Medicine, University of Belgrade, SERBIA

## Abstract

Follicular atresia is a cell death event that occurs in the great majority of follicles before ovulation in the mature mammalian ovary. Germ cell loss has been mainly associated to apoptosis although autophagy also seems to be at play. Aimed to increase our understanding on the possible cooperating role of autophagy and apoptosis in follicular atresia and/or follicular survival, we analyzed both programmed cell death mechanisms in a rodent model, the South American plains vizcacha, *Lagostomus maximus*. Female vizcacha shows highly suppressed apoptosis-dependent follicular atresia in the adult ovary, with continuous folliculogenesis and massive polyovulation. This strategy of massive ovulation requires a permanent remodeling of the ovarian architecture to maintain the availability of quiescent primordial follicles throughout the individual's reproductive lifespan. We report here our analysis of autophagy (BECN1, LAMP1 and LC3B-I/II) and apoptosis (BCL2 and ACTIVE CASPASE-3) markers which revealed interactive behaviors between both processes, with autophagy promoting survival or cell death depending on the ovarian structure. Strong BECN1, LC3B-II and LAMP1 staining was observed in atretic follicles and degenerating corpora lutea that also expressed nuclear ACTIVE CASPASE-3. Healthy follicles showed a slight expression of autophagy proteins but a strong expression of BCL2 and no detectable ACTIVE CASPASE-3. Transmission electron microscopy revealed a high formation of autophagosomes, autolysosomes and lysosomes in atretic follicles and degenerating corpora lutea and a low number of autophagic vesicles in normal follicles. The co-expression of LC3B-BECN1, LC3B-LAMP1 and LC3B-ACTIVE CASPASE-3 was only detected in atretic follicles and degenerating corpora lutea, while co-expression of BCL2-BECN1 was only observed in normal follicles. We propose that autophagy could act as a mechanism to eliminate altered follicles and remnant corpora lutea providing the necessary space for maturation of primordial follicles that continuously enter the growing follicular pool to sustain massive ovulation.

## Introduction

Follicular atresia is a cell death event that occurs in the great majority of follicles before ovulation in the mature mammalian ovary [1]. It can be attributed to a process of extreme control selection to preserve good quality oocytes and maximize reproductive success [2–4]. Type I programmed cell death (PCD), apoptosis, has been shown as the main responsible mechanism underlying germ cell loss through follicular atresia [5–7]. Nevertheless, it has been suggested that other concurrent mechanisms of PCD could be at play; among these, type II PCD, autophagy, has been implicated [2, 3, 8–13]. It has been previously found that both apoptosis and autophagy are involved in regulation of the follicle development as well as follicular atresia [14–19].

Autophagy is an evolutionarily conserved cellular process, from yeast to mammals, through which a cell degrades their own abnormal proteins, aggregates and organelles by the autophagosome [20–23]. This process is important for normal development, tissue/organ remodeling, and cell death/survival [24–26]. Recent reports provided evidence that autophagy is a lysosome-mediated degradation pathway in which fusion of double membrane autophagosomes and lysosomes are implicated (macroautophagy) [20]. The process involves the sequestration and transport of cytoplasmic material to the lysosome for degradation and recycling [25].

Basal levels of autophagy are of great importance for maintaining cellular homeostasis under natural conditions [26–28]. Autophagy can be triggered by different causes of environmental stress such as nutritional deficiencies [29], hypoxia [30], amino acid deprivation [31, 32], DNA or organelle damage [33, 34], high temperatures [35], reactive oxygen species [36] or even hormonal alterations [37, 38]. When the stimulus is exacerbated or if the damage is not reversed, autophagy ends up triggering cell death or activating apoptosis [39, 40]. Thus, autophagy could either promote cell death or protect cells from diverse types of injuries depending on cellular and environmental conditions [41]. In this context, apoptosis and autophagy have been proposed as cooperating mechanisms. However, how both processes interact in the mammalian ovary has not been thoroughly investigated [42].

Aimed to increase our understanding on the possible cooperating role of autophagy and apoptosis in follicular atresia and/or follicular survival, we analyzed both PCD mechanisms in an emerging seasonal breeding rodent model, the South American plains vizcacha, *Lagostomus maximus*. *L*. *maximus* is a fossorial hystricognathe distributed primarily in the Pampa plains of Argentina [43]. Females show highly suppressed apoptosis-dependent follicular atresia in the adult ovary, with continuous folliculogenesis and massive polyovulation that can reach up to 800 oocytes per estral cycle [44, 45]. This strategy of massive ovulation, comparable to other non-mammalian vertebrates, which seems to replace intraovarian germ cell loss seen in most mammals, requires a permanent remodeling of the ovarian architecture to maintain the availability of quiescent primordial follicles throughout the individual's reproductive lifespan [46–49]. We report here our analysis of main autophagy and apoptosis markers in the ovary of mature *L*. *maximus* females which reveals interactive behaviors between both processes, where autophagy could promote survival or cell death depending on the ovarian structure. We propose that autophagy could act as a mechanism to eliminate altered follicles and remnants of corpora lutea, providing the necessary space for maturation of primordial follicles that continuously enter the growing follicular pool to sustain massive ovulation.

## Materials and methods

### Animals

A total of 14 non-pregnant adult female plains vizcacha, *Lagostomus maximus*, were captured over 2 consecutive years during the non-breeding season using live traps located at the entrance of burrows from a natural resident population at the Estación de Cría de Animales Silvestres (ECAS), Ministry of Agriculture, Buenos Aires, Argentina. The number of animals captured was approved by the Ministry of Agriculture Authority of the Buenos Aires Government. Experimental protocols were approved by the Institutional Committee on the Use and Care of Experimental Animals (CICUAE-Universidad Maimónides, protocol number 0021/15). The handling and killing of animals was performed in accordance with the guidelines published in the National Institutes of Health (NIH) guide for the care and use of laboratory animals (National Research Council-USA, 2011). Animals were anaesthetized by the intramuscular administration of 13.5 mg/kg body weight of ketamine chlorhydrate (Holliday Scott S.A., Buenos Aires, Argentina) and 0.6 mg/kg body weight xylazine chlorhydrate (Richmond Laboratories, Veterinary Division, Buenos Aires, Argentina). Blood samples were taken by cardiac puncture. After bleeding, animals were sacrificed by an intracardiac injection of Euthanyl (0.5 ml/kg, Brouwer S.A., Buenos Aires, Argentina).

### Reproductive status

Reproductive status of adult non-pregnant non-ovulating females (n = 14) was assessed on the basis of the weight of animals (> 2.5 Kg), time of capture (February to March) according to the natural reproductive cycle described by Llanos and Crespo [52], our own previous field expertise [45, 46, 49–53] and the absence of embryos implanted in the uterine horns. Non-pregnant non-ovulating status was corroborated by circulating serum levels of LH, progesterone and estrogens determined as previously described [50]. Hormone levels were as follows: LH, 0,56 ng/ml ± 0,42 (radioimmunoassay); estradiol, 58,42 ± 16,56 pg/ml (DRG® Estradiol ELISA EIA-2693); progesterone, 0,50 ± 0,39 ng/ml (17-OH Progesterone ELISA EIA-1292) corresponding to previous reported values [50]. The ovulatory status was assessed by ovary naked-eye inspection for the presence of ovulatory stigmata at the time of sacrifice.

### Tissue collection and ovarian histology

Ovaries from non-pregnant, non-ovulating females were exposed and removed. One ovary was fixed in cold 4% neutral-buffered *para*-formaldehyde (PFA) for 24 h. PFA-fixed tissues were dehydrated in increasing graded alcohols, embedded in paraffin, serially sectioned at 6 μm, and mounted onto cleaned coated slides. Sections were dewaxed in xylene (Sigma Chemical Co., St. Louis, MO, USA) and re-hydrated in decreasing graded alcohols. At least 3 to 5 slides of each specimen were stained with haematoxilyn-eosin for general histology inspection of the ovulatory status and follicle development. Hematoxylin-eosin stained ovary sections were examined to determine the relative abundance of primordial, primary, secondary or pre-antral and antral (pre-ovulatory) follicles and primary corpora lutea (CL), as previously described [50, 51]. The remaining consecutive serial-sectioned slides were used for immunohistochemistry. The other ovary was cut into equal halves and processed for western blot analysis or transmission electron microscopy (TEM).

### Immunohistochemistry

Dewaxed and re-hydrated ovarian sections were treated with 3% H_2_O_2_ for 20 min at room temperature to block the activity of endogenous peroxidase. Sections were placed in sodium citrate buffer (10mM sodium citrate, 0.05% Tween-20, pH 6.0) for heat-induced epitope retrieval for 20 min in a water bath at 100°C, followed by incubation in a blocking solution containing 10% bovine fetal serum in PBS (pH 7.4) for 30 min at room temperature. Blocked sections were incubated overnight at 4°C with specific primary antibodies: rabbit anti-LAMP1 IgG (1:500, ab24170, Abcam, Cambridge, UK), rabbit anti-LC3B IgG (1:500, ab48394, Abcam, Cambridge, UK), rabbit anti-BECN1 IgG (1:500, ab62472, Abcam, Cambridge, UK) and rabbit anti-ACTIVE CASPASE-3 IgG (1:300, ab2302 and ab13847, Abcam, Cambridge, UK). Then, sections were incubated with secondary biotinylated anti-rabbit IgG (1:200, Vector Labs, Peterborough, UK) for 1h. After further washing in PBS, sections were incubated with avidin–biotin complex (ABC Vectastain Elite Kit, Vector Laboratories, Burlingame, CA, USA), and then incubated with 1:100 diluted streptavidin-peroxidase complex (ABC kit, Vector Labs, Peterborough, UK) for 30 min. The reaction was visualized with DAB (SK-4100, DAB Kit, Vector Laboratories, Burlingame, CA, USA). Microscope images of the immunorreactivity were captured with a light microscope (BX40, Olympus Optical Corporation, Tokyo, Japan), fitted with a digital camera (390CU 3.2 Megapixel CCD Camera, Micrometrics, Spain) and the image software Micrometrics SE P4 (Standard Edition Premium 4, Micrometrics, Spain). All images were taken the same day under the same light to avoid external variations. Staining for each antibody was repeated at least three times in separate assays for each specimen, using a minimum of two slides per assay. Proximal, medial and distal sections of the whole organ were used. All antibodies were screened in serial sections on the same slide. Brain, muscle and blood vessel tissue from *L*. *maximus* embryos were used as positive controls for BECN1, LC3B and LAMP1, respectively. As additional positive controls, testes of *L*. *maximus* [54] and laboratory mouse ovaries [16] were included. As negative control, normal rabbit serum was used instead of the primary antibody. No specific immunoreactivity was detected in these sections.

### Immunofluorescence

For protein colocalization analysis, dewaxed and re-hydrated sections were blocked with normal goat or rabbit serum (Vectastain Elite ABC Kit, Vector Laboratories, Burlingame, CA, USA) for 30 min at room temperature. Immunoreactivity was detected by incubating the slides overnight at 4°C with specific primary antibodies: rabbit anti-LAMP1 IgG (1:500, ab24170, Abcam, Cambridge, UK), rabbit anti-LC3B IgG (1:500, ab48394, Abcam, Cambridge, UK), rabbit anti-BECN1 IgG (1:500, ab62472, Abcam, Cambridge, UK), rabbit anti-ACTIVE CASPASE-3 IgG (1:300, ab2302 and ab13847, Abcam, Cambridge, UK), anti-goat BCL2 (1:500, Sc– 492 Santa Cruz Biotechnology, Inc. (California U.S.A). After further washing in PBS-Tween 20, slides were incubated with secondary antibodies Alexa-Fluor 488 coupled gout anti-rabbit IgG (Invitrogen Corp.), Alexa-Fluor 555 coupled donkey anti-rabbit IgG (Invitrogen Corp.) or Alexa- Fluor 488 coupled donkey anti-rabbit IgG (Invitrogen Corp.) for 1 h at room temperature. Sections were rinsed in PBS-Tween 20 and a second block of endogenous IgG was performed. Sections were incubated with the second primary antibody and then with secondary antibodies conjugated with a fluorochrome in a humid and dark chamber overnight at 4°C. Counterstaining with DAPI (100ng/ml in PBS) was performed for 20min at room temperature. Co-localization of proteins was visualized by immunofluorescence using a Nikon C1 Plus Laser microscope (Nikon Inverted Research Microscope Eclipse Ti, Nikon Corp., Tokyo, Japan) and images were analyzed with the EZ-C1 software (EZ-C1 Software v3.9, Nikon Ltd., London, UK).

### Quantification of autophagic and apoptotic markers in ovarian structures

The quantification of positive follicles and corpora lutea for BECN1, LAMP1, LC3B and A-C3 was performed by counting all positive and negative structures for each marker in a total of three sections per animal from randomly-chosen slides. Follicles with immunreactive oocyte and Granulosa cells for each protein were considered as positive. An average of 500 primordial, 200 primary, 200 secondary, 20 antral, 15 atretic follicles and 30 corpora lutea were counted in each case. Since LC3B-II localizes to the autophagosomes, it can be visualized and quantified by counting LC3B puncta with optical microscopy [55]. Then, the quantification of the number of puncta in each LC3B positive ovarian structure that represents LC3B-II was calculated as the average count of puncta per oocyte or luteal cell visualized. Reactive structures were counted in immunohistochemistry-treated sections using an Olympus BX40 microscope (Tokyo, Japan) at 400X magnifications. Double counting was performed independently by two observers.

### Transmission electron microscopy (TEM)

In order to analyze ultracellular autophagic vacuoles, ovaries were sectioned in blocks of 1x1 mm thick with a scalpel blade under an Olympus SZX7 stereomicroscope, fixed in cold 4% paraformaldehyde/0.25% glutaraldehyde in 0.1 M phosphate buffer (pH 7.4) during 72h, and then transferred to fresh phosphate buffer. Sections were subsequently post fixed in 2% osmium tetroxide containing 1.5% potassium ferricyanide for 1 h, dehydrated in graded alcohols and propylene oxide and embedded in Fluka Durcupan (Sigma). Semithin sections (0.50μm thick) were obtained using an ultramicrotome (Reichert Jung Ultracut E) and counterstained with toluidine blue for observation in a light microscope. The area of interest was selected and ultrafine cuts (70-90nm thick) were made with an Ultramicrotome (Reichert Jung Ultracut E). The cuts were mounted on copper grids and contrasted with uranyl acetate and lead citrate (Reynolds method). The cuts were observed in the Zeiss 109 transmission electron microscope attached to the Gatan 1000 digital camera.

### Statistical analysis

Statistics were performed by using one-way ANOVA followed by Bonferroni post-hoc test, in order to compare the level of each protein expression in the different ovarian structures analyzed. InfoStat 2010 software (www.infostat.com.ar) was used and differences were considered significant when p<0.05.

## Results

### Autophagy-related proteins in the ovary of vizcacha

We analyzed the expression of two autophagy-related proteins: i) BECLIN 1 (BECN1) involved in the formation of the pre-autophagosomal structure, and ii) microtubule-associated protein 1 light chain 3 (LC3B) which is cleaved or converted from LC3B-I to LC3B-II during autophagy induction. LC3B-II then becomes localized to isolated membranes and autophagosome membranes indicating that autophagy is indeed occurring. Thus, the amount of LC3B-II correlates with the number of autophagosomes [27].

**Fig 1:**
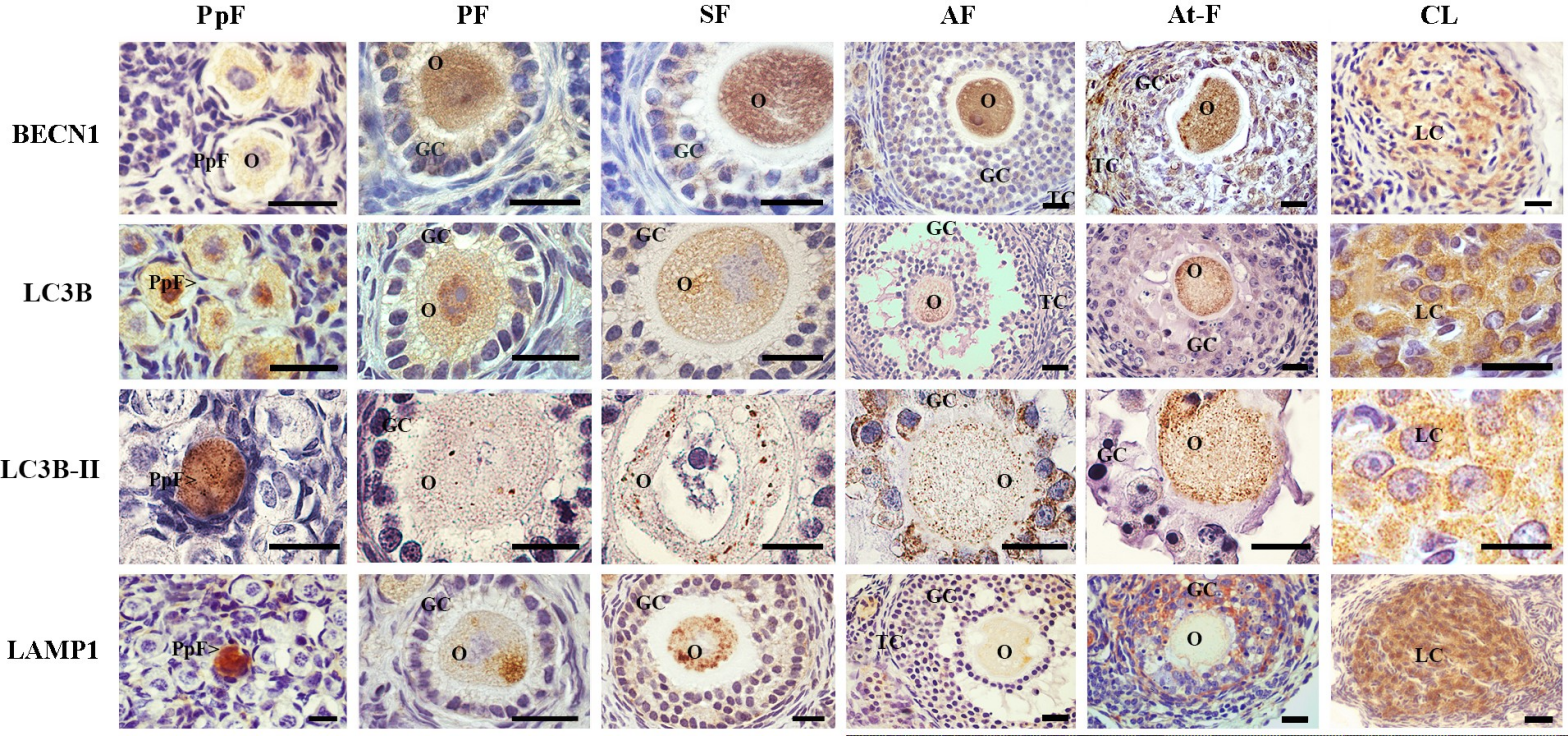
Immunolocalization of autophagy-related proteins in the ovary of *L*. *maximus*. Immunostanning of BECN1, LC3B and LAMP1 in primordial, primary, secondary, antral and atretic follicles and corpora lutea. Note the punctuate pattern of LC3B-II in oocytes of all ovarian structures. PpF: Primordial follicle, PF: primary follicle, SF: secondary follicle, AF: antral follicle, At-F: atretic follicles, CL: Corpora lutea, O: oocyte, GC: granulosa cells, TC: theca cells and LC: luteal cells. Scale bar: 40 μm (short) and 100 μm (large).

BECN1 was immunolocalized both in the cytoplasm of oocytes and granulosa cells in primordial, primary and secondary follicles (Fig 1). No expression of BECN1 was detected in theca cells of immature follicles (Fig 1). BECN1 was weakly immunolocalized in oocyte, granulosa cells and theca cells from mature antral follicles (Fig 1). In turn, strong BECN1 immunostaining was observed in granulosa cells of atretic follicles and in luteal cells of degenerating corpora lutea (Fig 1). The percentage of BECN1 expression increased in primary, secondary, antral and atretic follicles respect to primordial follicles (p<0.05, Table 1). The majority of corpora lutea (92,66%) were positive for BECN1, being this structure the one with the greatest expression of this protein (p<0.05, Table 1).

**Table 1:** Ovarian structures immunorreactive for autophagic-related proteins and ACTIVE CASPASE-3 in *Lagostomus maximus*.

	PpF*	PF	SF	AF	At-F	CL
**BECN1**	24,28±2,61^a^	39,46±7,66^c^	56,81±7,04^b^	52,59±7,02^b^	39,01±6,19^c^	92,66±6,52^d^
**LC3B**	5,42±0,83^a^	19,74±3,70^b^	23,26±4,93^b,c^	31,05±7,16^c^	76,91±8,50^d^	84,85±5,86^d^
**LAMP1**	37,73±0,99^a,c^	47,12±6,66^c^	36,60±4,31^a^	45,74±3,55^a,c^	86,70±6,96^b^	92,38±3,29^b^
**A-C3**	3,28±0,35^a^	10,69±5,49^a^	11,66±6,10^a^	9,58±6,45^a^	91,00±5,86 ^b^	76,95±11,21^c^

*PpF, primordial follicle; PF, primary follicle; SF, secondary follicle; AF, antral follicle; At-F, atretic follicle; CL, corpora lutea. Values are expressed as mean percentage ± SEM. Different letters in each row indicate only significant differences (p<0.05) for each protein between the ovarian structures analyzed.

The expression of LC3B was restricted to the cytoplasm of oocytes from primordial, primary, secondary, antral and atretic follicles and corpora lutea (Fig 1). Nuclear expression of LC3B was observed in oocytes from few primordial and primary follicles (Fig 1). A small proportion of primordial follicles (5,42%) were positive for LC3B (Table 1). Once primordial follicles entered the growing pool (primary to antral stages), mean percentage of LC3B detection increase to 20–30% (p<0.05, Table 1). On the other hand, in degenerative structures (atretic follicles and regressing corpora lutea), mean percentage of LC3B detection significantly raised up to 75–85% respect to healthy follicles structures (p<0.05, Table 1). LC3B-I showed, as expected, a homogeneous distribution both in cytoplasm and nucleus whereas LC3B-II displayed a punctuated signal (Fig 1). LC3B-II puncta per oocyte were counted in normal and atretic follicles and luteal cells as well. Low numbers of LC3B-II puncta/cell were found in primordial (6.55 ± 2.54) and primary (5.63 ± 1.93) follicles, notably increasing in growing secondary (16.07 ± 8.93) and antral (33.6 ± 2.2) follicles. The highest detection of LC3B-II puncta/cell was found in atretic follicles (149.33 ± 40.24) and corpora lutea (103 ± 8.21).

Finally, we evaluated the expression of LAMP1, the membrane protein of functional lysosomes. LAMP1 showed a homogeneous distribution in a few primordial follicles. Cytoplasmic LAMP1 with heterogeneous distribution was found in primary and secondary follicles; a slight signal was detectable in antral follicles (Fig 1). No expression of LAMP1 was detected in theca cells regardless of the ovarian structure (Fig 1). Most atretic follicles and luteal cells in corpora lutea showed strong LAMP1 signal (Fig 1). Estimation of the mean percentage of LAMP1-immunoreactive ovarian structures revealed a >30% abundance throughout folliculogenesis (Table 1). Atretic follicles and corpora lutea presented the highest expression of LAMP-1 compared to healthy follicles structures (p<0.05, Table 1).

### Autophagic flux analysis in the adult ovary of *L*. *maximus*

**Fig 2:**
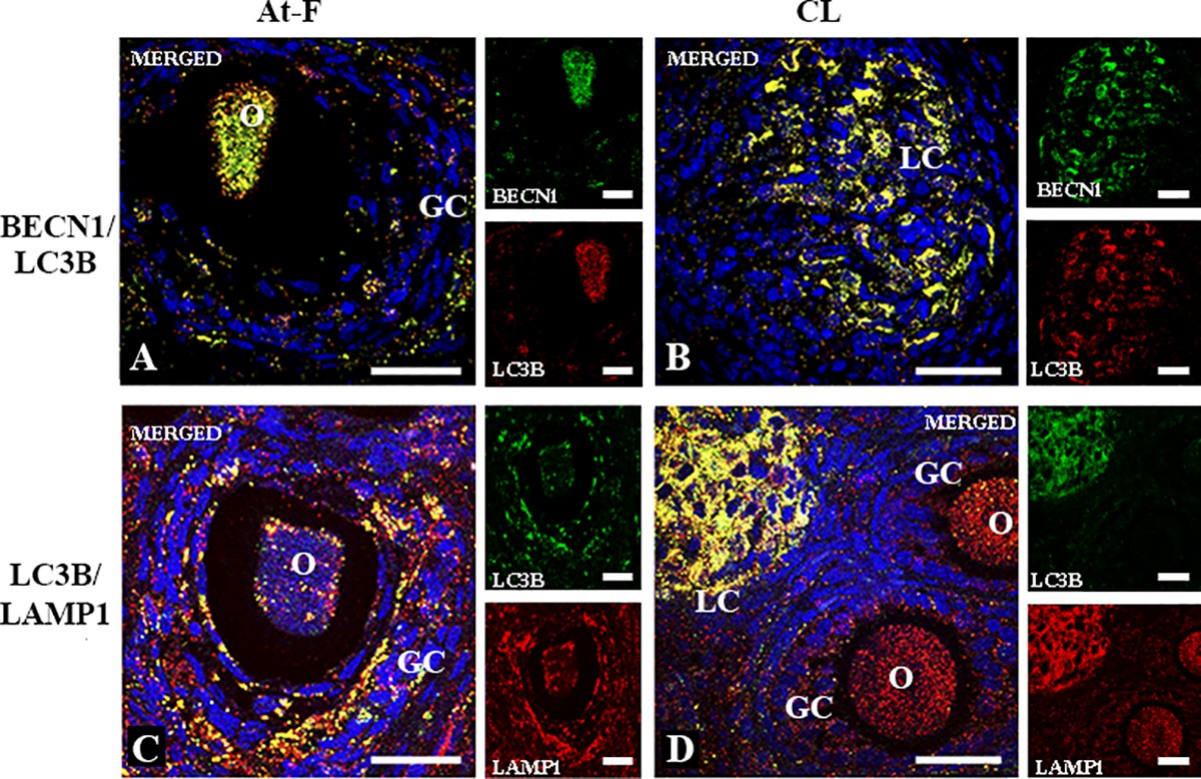
Two-color immunofluorescence of BECN1, LC3B and LAMP1 in atretic follicles and corpus luteum of *L*. *maximus*. Merged immunofluorecense images of BECN1 (cytoplasmic green staining) and LC3B (cytoplasmic red staining) in atretic follicles (A) and corpus luteum (B). Merged immunofluorecense images of LC3B (cytoplasmic green staining) and LAMP1 (cytoplasmic red staining) in atretic follicles (C) and corpus luteum (D). Note the simultaneous expression (citoplasmic yellow staining) of BECN1/LC3B and LC3B/LAMP1 in granulosa cells and oocytes from atretic follicles (A, C) and luteal cells of corpus luteum (B, D). All images show DAPI nuclear staining (blue). O: oocyte, GC: granulosa cells, LC: luteal cells. Scale bar, 60 μm.

Autophagic flux represents the dynamic process of autophagosome generation, their fusion with lysosomes, and the degradation of autophagic substrates in autolysosomes [21]. Therefore, we analyzed the co-localization of the autophagic substrates BECN1/LC3B (autophagosome generation) and LC3B/LAMP1 (fusion with lysosomes) to verify that these proteins were within the same cell as an indicator of the autophagic flux. We observed that LC3B co-localized both with BECN1 (Fig 2A and 2B) and with LAMP1 (Fig 2C and 2D) in oocytes and granulosa cells from atretic follicles and luteal cells. LC3B immunostaining showed the typical punctuated signal in all structures analyzed (Fig 2). No simultaneous expression of BECN1/LC3B and LC3B/LAMP1 was detected in primordial, primary and secondary follicles (Fig 2D).

### ACTIVE CASPASE 3 expression in the adult ovary of *L*. *maximus*

**Fig 3:**
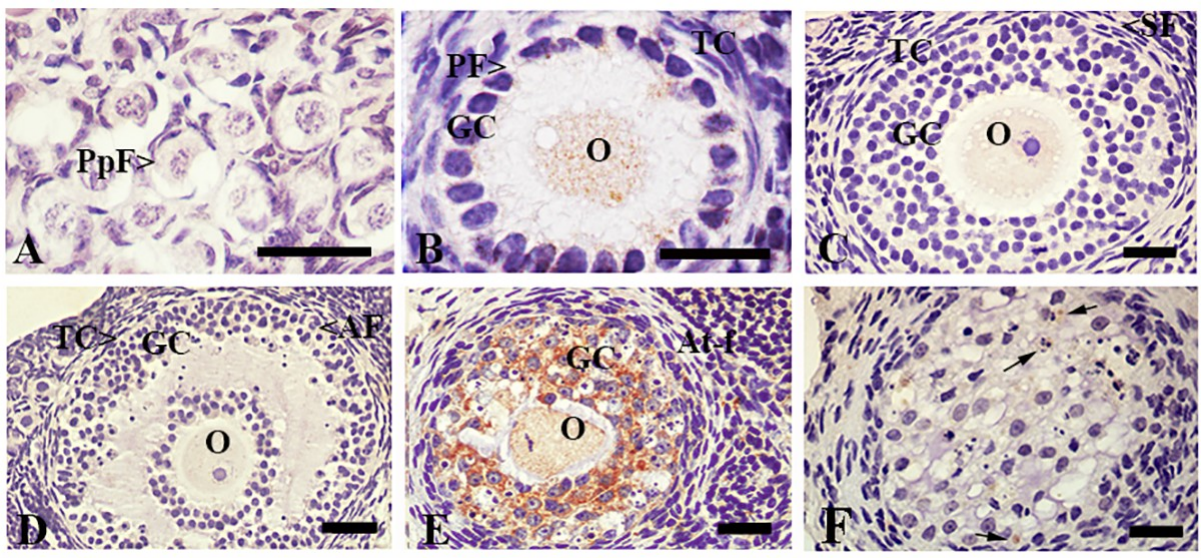
Immunolocalization of ACTIVE CASPASE 3 in the ovary of *L*. *maximus*. Immunostanning of ACTIVE CASPASE 3 in primordial (A), primary (B), secondary (C), antral (D) and atretic follicles (E) and corpora lutea (F). Note that granulosa cells of atretic follicles and luteal cells of corpora lutea showed strong staining for ACTIVE CASPASE 3. PpF: Primordial follicle, PF: primary follicle, SF: secondary follicle, AF: antral follicle, At-F: atretic follicles, CL: Corpora lutea, O: oocyte, GC: granulosa cells, TC: theca cells and LC: luteal cells. Black arrows indicate apoptotic cells. Scale bar: A, B, 40μm; C-F, 100μm.

The adult ovary of the vizcacha shows diminished apoptosis-mediated follicular atresia as a consequence of an over-expression of BCL2 protein [45]. In order to correlate autophagy and apoptosis, we analyzed the expression of ACTIVE CASPASE 3 (A-C3) as a late marker of apoptosis. The expression of A-C3 was mainly detected in atretic follicles and in a few cells from degenerating corpora lutea (Fig 3E and 3F). A-C3 was not detected in the majority of primordial, primary, secondary and antral follicles (Fig 3A–3D). Estimation of the mean percentage of A-C3-immunoreactive ovarian structures revealed low abundance (3 to 11%) throughout folliculogenesis (Table 1). Atretic follicles and corpora lutea presented the highest expression of A-C3 compared to healthy follicles structures (p<0.05, Table 1). Furthermore, the expression of A-C3 was higher in atretic follicles respect to corpora lutea (p<0.05, Table 1).

### Autophagy as a mechanism of cell survival or cell death in the adult ovary of *L*. *maximus*

**Fig 4:**
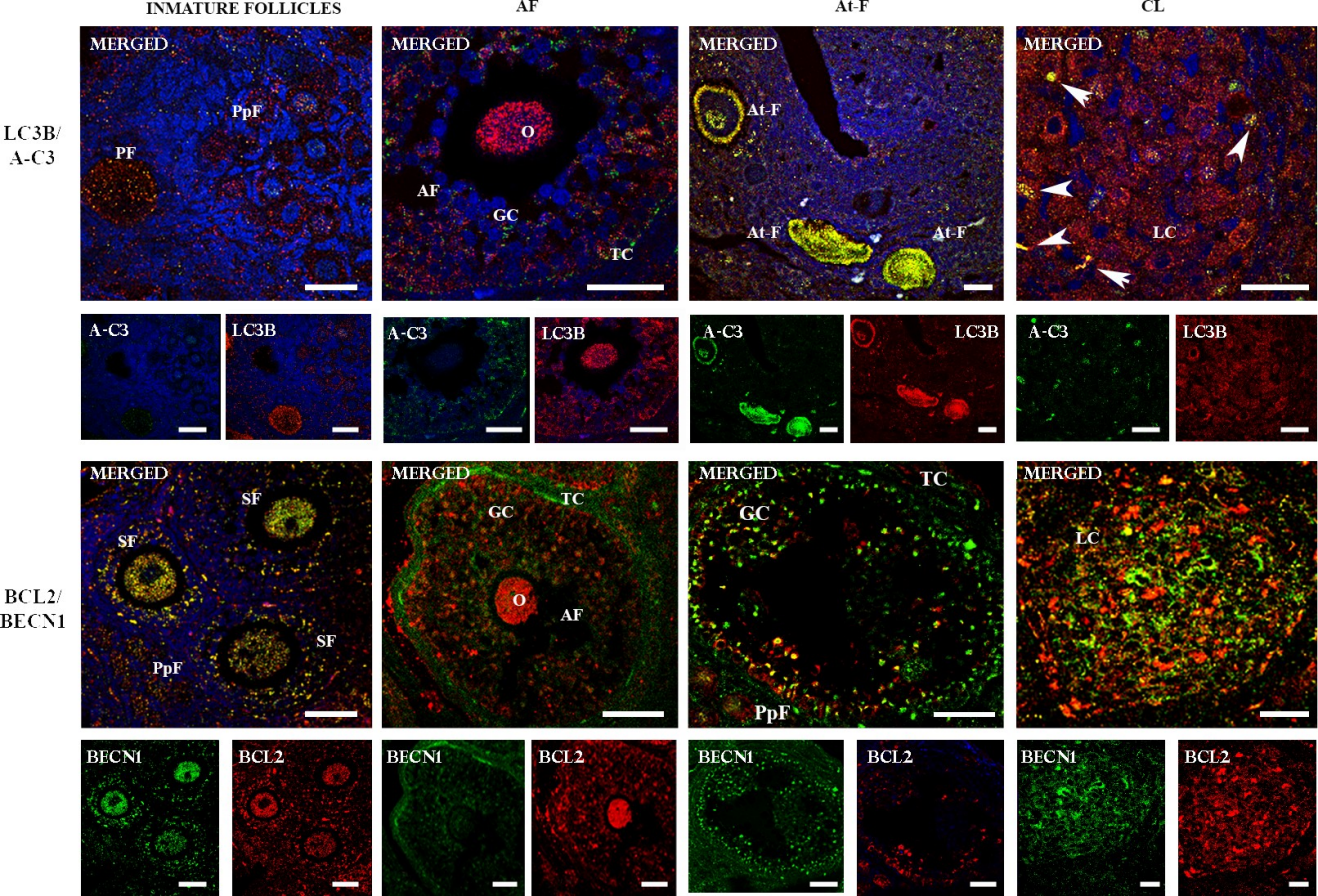
Two-color immunofluorecense of LC3B/ACTIVE CASPASE 3 and BECN1/BCL-2 proteins in the ovary of *L*. *maximus*. Merged immunofluorecense images of LC3B (cytoplasmic red staining) /ACTIVE CASPASE 3 (cytoplasmic and nuclear green staining) and BECN1 (cytoplasmic green staining) / BCL2 (cytoplasmic red staining) in immature, antral and atretic follicles and corpus luteum. Note the simultaneous expression (cytoplasmic yellow staining) of LC3B/ACTIVE CASPASE 3 and BECN1/BCL2 in atretic follicles and immature follicles, respectively. O: oocyte, GC: granulosa cells, TC: theca cells and LC: luteal cells, A-C3: ACTIVE CASPASE 3. White arrows indicate co-localization of A-C3 and LC3B in luteal cells. Scale bar: 40 μm (short) and 60 μm (large).

In order to analyze the apoptotic process in follicles and corpora lutea showing autophagic flux, we investigated the co-localization of A-C3 and LC3B. The simultaneous expression of A-C3 and LC3B was detected in oocytes and granulosa cells in atretic follicles (Fig 4). On the other hand, LC3B was detected in the majority of the luteal cells and a few LC3B-positive luteal cells showed A-C3 signal (Fig 4). Primordial, primary, secondary and antral follicles did not show simultaneous expression of LC3B/A-C3 (Fig 4).

Then, we co-localized anti-apoptotic BCL2 and BECN1 proteins to evaluate cell survival in all follicular stages and corpora lutea. The simultaneous expression of BECN1 and BCL2 was observed in oocytes and granulosa cells of primordial, primary, secondary and early antral follicles (Fig 4). Although BCL2 and BECN1 were expressed in mature antral follicles and corpora lutea, no co-localization of both proteins was observed (Fig 4). In this case, BCL2 was detected in granulosa cells and oocyte while BECN1 was detected in theca cells (Fig 4). In atretic follicles, BCL2 was detected in isolated granulosa cells while BECN1 was detected in the majority of granulosa and theca cells and oocytes (Fig 4).

### Ultrastructural features of autophagy and apoptosis in the ovary of vizcacha

**Fig 5:**
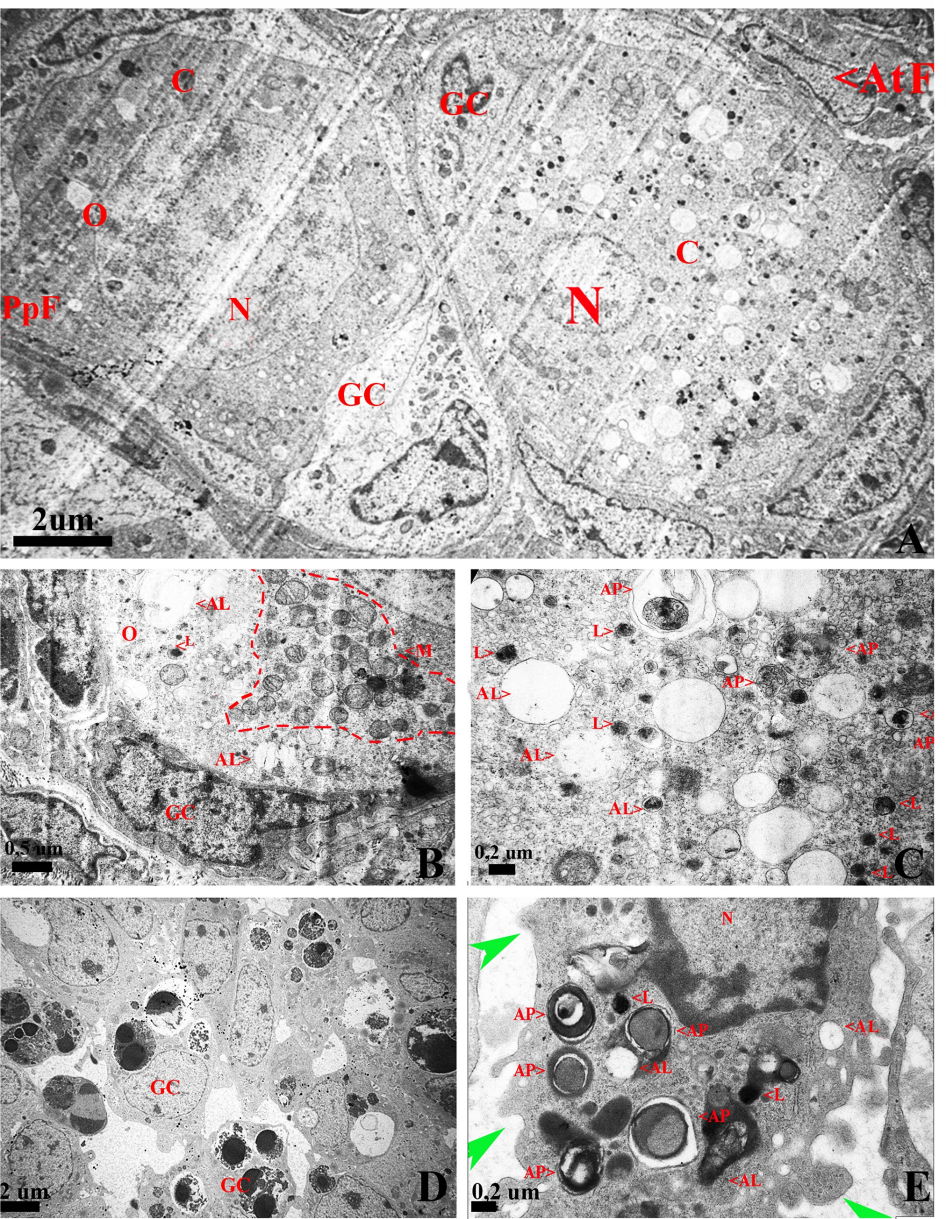
TEM images of ultrastructural features of autophagy and apoptosis in the ovary of *L*. *maximus*. (A) Normal primordial follicle with homogeneous chromatin in the nucleus and atretic follicle with altered morphology characterized by involuted nucleus, condensation of chromatin and numerous vesicles in the cytoplasm. (B) Detail of the normal primordial follicle cytoplasm containing a large amount of mitochondria (red dotted line) and few autolysosomes. (C) Detail of the altered oocyte with numerous autophagosomes and autolysosomes. (D) Apoptotic granulose cells from atretic follicles characterized by condensed nuclei, apoptotic bodies and (E) plasmatic membrane blebs (green arrow), autophagosomes, autolysosomes and lysosomes. N: nucleus, C: cytoplasm, M: mitochondria, AP: autophagosome, AL: autolysosome, L: lysosomes, PpF: primordial follicle, AtF: atretic follicle. GC: granulosa cell.

Transmission electron microscopy (TEM) was used to determine the presence of autophagosomes, autolysosomes and lysosomes in atretic and healthy follicles. TEM showed differences between oocytes from atretic and healthy follicles. Oocyte in healthy follicles maintained a close relation with the somatic cells, their cytoplasm was homogeneous, mitochondria almost covered the entire cytoplasm and chromatin was sparsely distributed in the nucleoplasm (Fig 5). Healthy oocytes were characterized by the presence of a small number of autophagic vesicles (Fig 5A and 5B). On the contrary, oocytes in atretic follicles displayed abundant autophagic vesicles containing highly degraded cytoplasmic material at different stages of maturation (Fig 5). Double membrane autophagosomes with cytoplasmic material, single membrane autolysosomes with dark and small cytoplasmic remains and also many lysosomes, were found in oocytes in atretic follicles (Fig 5). Granulosa cells from atretic follicles showed the typical ultra-structural features of apoptosis: condensed nuclei, apoptotic bodies and blebbing of cytoplasmic membrane (Fig 5D). Moreover, granulosa cells showed large numbers of autophagic vesicles containing cytoplasmic material at different stages of degradation (Fig 5E).

**Fig 6:**
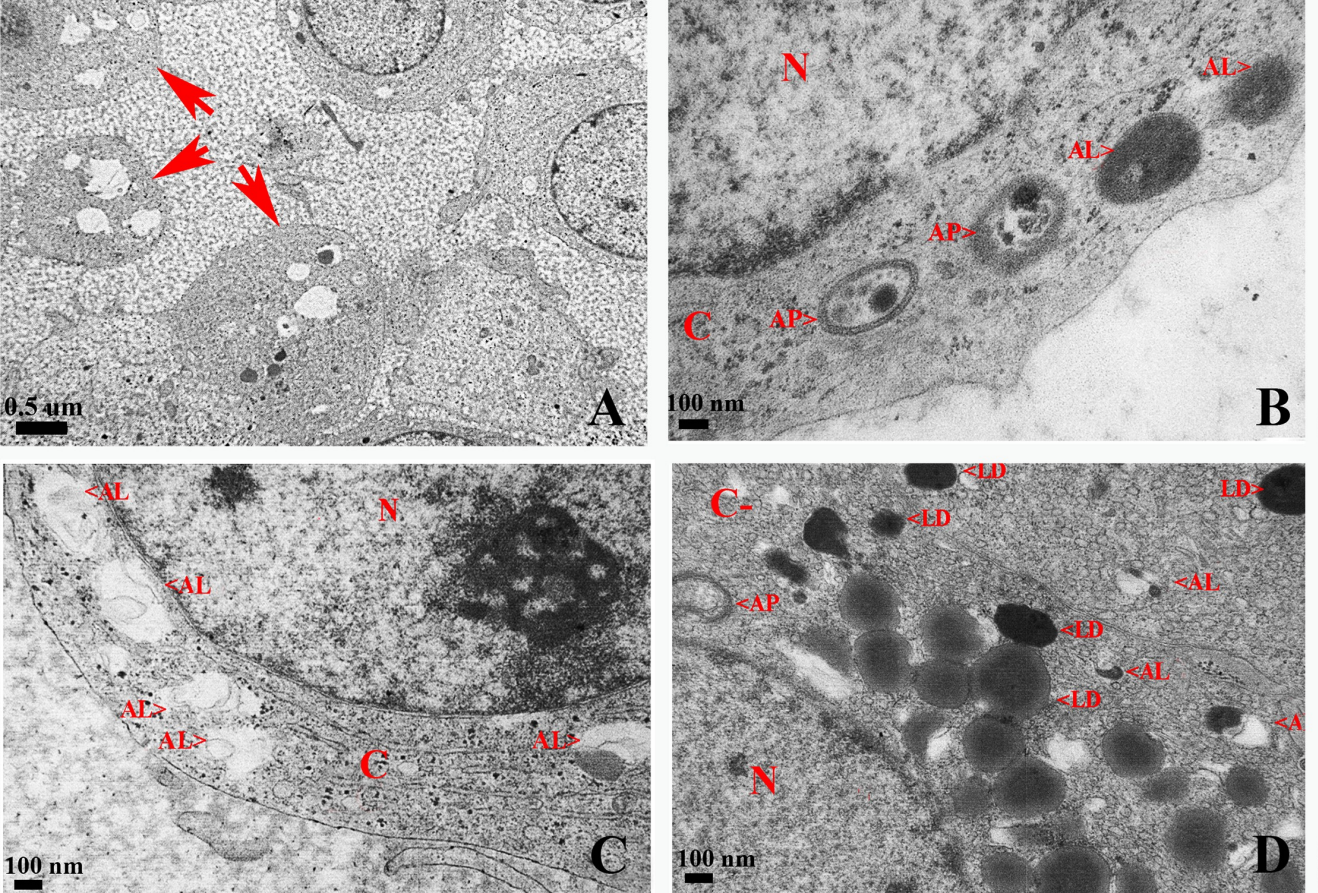
TEM images of ultrastructural features of autophagy in luteal cells of degenerative corpus luteum in the ovary of *L*. *maximus*. Representative images of luteal cells with altered morphology (A, red arrows), autophagosomes and autolysosomes in different stages of maturation (B), large quantity of autolysosomes (C) and numerous lipid drops (D). N: nucleus, AP: autophagosome, AL: autolysosome, L: lysosome, LD: lipid drops, C: cytoplasm.

TEM analysis in corpus luteum showed that most luteal cells presented an altered morphology characterized by loss and deformation of the rounded cell structure, loss of cell contact, absent nucleus and vacuolization and accumulation of lipofuscin pigments (Fig 6A). Interestingly, luteal cells did not show apoptotic characteristics but a large quantity of autophagic structures was observed (Fig 6B). Also, luteal cells showed many lipid drops, abundant autophagosomes, autolysosomes and lysosomes scattered in the cytoplasm (Fig 6C and 6D).

## Discussion

In the last years, autophagy has gained relevance as a mechanism controlling tissue homeostasis, acting as an alternative death pathway to apoptosis or as a mechanism of adaptation to stress to avoid cell death in the mammalian life cycle [56, 57]. The involvement of autophagy and its interaction with the apoptotic process in the ovary has been poorly investigated. Here, using *L*. *maximus* females as a model of poly-ovulation and continuous folliculogenesis, we provide evidence that autophagy could act as a dual mechanism to maintain cell survival from primary to antral follicle or act as a cell death mechanism in atretic follicles and degenerating corpora lutea.

In order to maintain poly-ovulation, *L*. *maximus* females continuously recruit primordial follicles to enter the growing follicular pool [44–47]. In this scenario, the continuous entrance of primordial follicles into the growing pool and the highly diminished rate of apoptosis-induced follicular atresia generate a need for nutrient supply and energy to maintain cell homeostasis and follicular survival. Here, we showed that autophagy may be an important regulator of germ cell survival prior to the formation of the follicular pool since the simultaneous expression of BECN1 and BCL2 and the presence of few autophagic vacuoles were observed. In this context, *Gawriluk et al* [16] showed that knocking-out *Beclin1* resulted in an over loss of oocytes, suggesting that autophagy acts as a cell survival mechanism during germ cell loss and oocyte survival in the mouse ovary [16].

Different stress conditions can trigger autophagy and apoptosis within a single cell and they have been shown as cooperating mechanisms that, depending on the cellular context, can determine cell death or survival [22, 26–28, 40]. The relationship between autophagy and apoptosis is coordinated by the interaction of the BH3 binding domains of BECN1 and BCL2 [58, 59]. Here, the co-expression of BECN1 and BCL2, together with the low levels of A-C3 in early follicular maturation stages, suggests the involvement of autophagy in maintaining cellular homeostasis in the ovary of *L*. *maximus*. Moreover, healthy oocytes were characterized by scarce expression of LC3B-II, with small quantities of autophagic vesicles. In line with our results, previous reports showed low levels of autophagic-related proteins in healthy follicles in mouse and rat ovaries [2, 7, 13, 14, 16, 26]. In this context, it has been proposed that in basal levels of autophagy (pro-survival), the interaction between BECN1 and BCL2 occurs; however, the dissociation of the complex leads to an increase in autophagy, which can trigger cell death depending on the cellular context [57]. In *L*. *maximus*, we have previously shown a high expression of BCL2 in all follicular stages [44, 45]; thus, this can avoid BECN1/BCL2 dissociation to maintain follicular survival.

In most mammals, the majority of follicles undergo atresia during folliculogenesis [7]. Although the presence of autophagic-related proteins in atretic follicles has been reported [18], it seems well established that follicular atresia is due to the apoptosis of granulosa cells involved in the synthesis of molecules that are essential for follicular and oocyte growth [60]. Recently, *Escobar et al*. [18] reported that a significant number of rat oocytes express LC3, LAMP1 and A-C3 and show features of both apoptosis and autophagy. Moreover, it was proposed a combined role of apoptosis and autophagy in the elimination of immature rat oocytes through the expression of LC3B and A-C3 [15, 18]. Here, we demonstrated for first time that atretic follicles show autophagic flux due to the simultaneous expression of the autophagic related-proteins BECN1, LC3B-II and LAMP1 as well as the presence of abundant autophagosomes and autolysosomes with degraded cytoplasmic material and lysosomes within the cytoplasm. We also detected the simultaneous expression of LC3B-II and A-C3 in oocytes and granulosa cells with both apoptotic and autophagic morphological features, suggesting a concurrent activity of these mechanisms in the same cell. Recent studies suggested that autophagy induces apoptosis by triggering the accumulation of autophagosomes in granulosa cells [15, 61–66]. Moreover, we did not observe simultaneous expression of BECN1 and BCL2 in atretic follicles, possibly related to the loss of the interaction between both proteins that induce autophagy and promote cell death [57]. Based on our results in *L*. *maximus* and previous reports [44, 45], we suggest that granulosa cells die by apoptosis and/or autophagy. Thus, death of granulosa cells may deprive of nutrients the oocyte which then becomes susceptible to die by autophagy.

Concerning the regression of corpora lutea, several authors have proposed apoptosis as the mechanism involved in luteal cell death [65]. However, the apoptotic process in corpora lutea is controversial when different mammal species are analyzed. Thus, apoptosis might not be the only mechanism involved in corpora lutea regression. Few studies have investigated the involvement of autophagy in corpora lutea regression in mammals [67]. Recently, it has been reported that the characteristics of morphological regression of corpora lutea indicate that luteal cell death is induced by autophagy in primate ovaries [68–69]. Here, we reported the presence of autophagic flux and autophagosomes, autolysosomes and lysosomes within the cytoplasm of luteal cells. On the other hand, the scarce expression of A-C3 suggests that the induction of autophagy could not be related to luteal cell apoptosis. Interestingly, it has been proposed that low levels of progesterone induce luteal cell death by autophagy due to the loss of the interaction between BECN1 and BCL2 [70–72]. In agreement with this, *L*. *maximus* females presented low serum levels of progesterone with respect to pregnant females [50].

## Conclusions

During *L*. *maximus* pregnancy, a process of pseudovulation that adds a considerable number of secondary corpora lutea, takes place at mid-gestation. Added secondary corpora lutea, thought to supply the progesterone necessary for the pregnancy to come to term, occupy most of the ovarian tissue [45, 50, 53]. Following parturition and lactation, the pre-ovulatory ovary shows scarce corpora lutea and no albicans bodies [45, 53]. In this scenario, the joint analysis of autophagy/apoptosis in the mature ovary of *L*. *maximus* lead us to propose that autophagy-induced death could act as a cleaning mechanism of cellular debris and cells that are no longer necessary, i.e. atretic follicles and corpora lutea, providing the necessary space for the formation and maturation of new follicles entering the growing follicular pool in order to sustain the massive ovulation that characterizes the ovary of *L*. *maximus*.
